# SG-APSIC1177: Improving the quality of sterile medical devices to international standards

**DOI:** 10.1017/ash.2023.114

**Published:** 2023-03-16

**Authors:** Nanthipha Sirijindadirat

**Affiliations:** President, Central Sterilizing Services Association, Bankok, Thailand

## Abstract

**Objectives:** The central sterile services department (CSSD) offers a variety of procedures, including collecting contaminated medical equipment from various agencies through a process of washing, cleaning, packing, and sterilization, followed by storage and distribution to internal and external departments. Plans to expand and open service to the Center of Excellence continue, which will result in a rapid increase in the processing of specialized medical devices. The Center of Excellence is a source of education, learning, teaching, and practice in the cleaning, disposal, and sterile treatment of medical devices. We sought to improve the quality of sterile medical equipment to satisfy international standards of safety. **Methods:** We implemented a 7-stage process in the CSSD and we developed a checklist with 8 categories of environmental development based on APSIC guidelines. We provided knowledge to CSSD employees, developed their skills, and promoted attitudes in various areas, including sterile work standards, the use of sterilization machines, and infection prevention and control both inside and outside the facility. **Results:** We established inventory control systems, storage guidelines, and disbursement of medical supplies and equipment in the CSSD. To improve machine use in sterilization treatment or disposal, we educated staff and provided practice of machine skills according to instructions for use. Information technology (IT) was used for the distribution process, medical device identification, recording sterilization, and statistical logging of sterilization and sterile services. **Conclusions:** Overall, we improved the quality of CSSD services and knowledge and practice of CSSD staff, and we achieved compliance with international standards. These measures ensure the safety of service providers and patients and build trust in the quality of CSSD services and output.

